# Chronic Anaphylaxis With Indolent Systemic Mastocytosis: A Case Report

**DOI:** 10.1155/crh/9562195

**Published:** 2025-11-06

**Authors:** Sarah Worth, Tracy I. George, Daniel J. Shaheen, Maria Roche, Pankit Vachhani

**Affiliations:** ^1^Department of Pharmacy, University of Alabama, Birmingham, Alabama, USA; ^2^ARUP Laboratories, Salt Lake City, Utah, USA; ^3^Blueprint Medicines Corporation, Cambridge, Massachusetts, USA; ^4^Department of Medicine, Division of Hematology/Oncology, University of Alabama at Birmingham, Birmingham, Alabama, USA

**Keywords:** allergy, anaphylaxis, avapritinib, case report, fulminant presentation, immunology, indolent, midostaurin, systemic mastocytosis

## Abstract

Systemic mastocytosis is a rare, clonal mast cell disease neoplasm driven by the *KIT* D816V mutation in greater than 95% of cases. The complex clinical presentation of systemic mastocytosis can make diagnosis challenging. Treatment strategies often focus on management of symptoms, but many patients' symptoms are not well controlled on these regimens and have poor quality of life. We report the case of a 35-year-old Caucasian female who suffered repeated life-threatening anaphylactic episodes that greatly decreased her quality of life and that the best supportive care measures failed to control. Following extensive diagnostic evaluations including physical examination, clinical labs, hematology, and next-generation genetic screening, indolent systemic mastocytosis was confirmed by bone marrow biopsy according to World Health Organization 2016 criteria. The patient was subsequently treated with avapritinib, a selective tyrosine kinase inhibitor, after which she reported improved quality of life and physical activity. The diagnosis and optimal management of this complex and rare disease require a multidisciplinary approach combined with effective personalized therapeutic strategies. A confirmed diagnosis of indolent systemic mastocytosis led to the patient receiving an approved targeted treatment, with a favorable outcome. Novel precision therapies and refined diagnostic guidelines are critical to meet the high unmet needs of patients with indolent systemic mastocytosis and improve their quality of life.

**Trial Registration:**

ClinicalTrials.gov identifier: NCT03731260

## 1. Introduction

Mastocytosis broadly describes a heterogeneous group of neoplasms characterized by uncontrolled proliferation and activation of mast cells (MCs) [1]. MCs accumulate and aggregate into organ systems including the skin, bone marrow (BM), gastrointestinal tract, lymph nodes, and other organs [1]. The clinical presentation is highly varied, ranging from isolated skin (cutaneous mastocytosis) to multisystemic involvement (systemic mastocytosis [SM]) [1]. Based on clinical, molecular, and pathological features, SM is further divided into indolent SM (ISM), BM mastocytosis, smoldering SM, SM with associated hematologic neoplasm, aggressive SM, and MC leukemia [1]. The prevalence of SM has been estimated at up to 1 in 5000 people [2–5], and ISM accounts for approximately 85% of cases of SM [4, 6, 7]; the incidence of ISM remains difficult to define.

Patients with ISM exhibit symptoms primarily related to MC degranulation, MC mediator release, and allergies or anaphylaxis [8, 9]. The *KIT* D816V mutation is the primary driver of SM pathogenesis and is present in > 95% of all adults with ISM and aggressive SM [10–12]. These patients can have lifelong debilitating symptoms across multiple organ systems and often rely on polypharmacy in an attempt to manage symptomatology. Despite multiple symptom-directed medications, many ISM patients' disease is not well controlled on their current regimens. The rarity of ISM, combined with its diverse, nonspecific clinical presentation, poses a serious diagnostic challenge, and patients must be seen by different medical specialists, depending on prominent symptoms [13]. Diagnosis of SM is only confirmed when at least 1 major and 1 minor or at least 3 minor criteria outlined by the World Health Organization (WHO) classification are met (Table 1) [14–16].

We report a case of a patient with repeated episodes of life-threatening anaphylaxis and other symptoms that greatly impacted her quality of life. Inadequate relief from best supportive care (BSC) available necessitated precision medicine-based approaches.

## 2. Case Presentation

**Figure 1:**
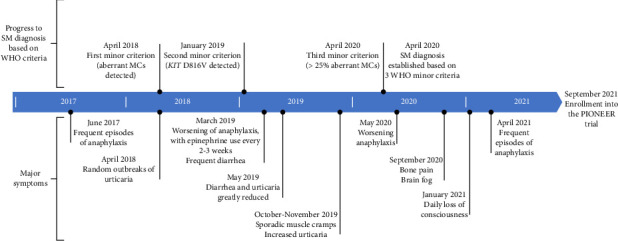
Timeline depicting major symptoms and SM diagnosis. MC, mast cell; SM, systemic mastocytosis; WHO, World Health Organization.

A 35-year-old Caucasian female presented to the emergency department (ED) in June 2017 with intense pain, dizziness, flushing, tachycardia, sensation of hotness, metallic taste in her mouth, convulsions, and loss of consciousness after a Hymenoptera (red wasp) sting. She went into cardiac arrest, was given adrenaline, and remained in intensive care for a week, where she was diagnosed with anaphylaxis. Prior to this episode, she was generally in good health and had no pertinent medical or family history. Thereafter, she reported random flushing episodes that persisted for several months. By December 2017, she was having allergic reactions, including sensations of angioedema and occasional anaphylaxis, due to new triggers, such as specific foods, perfumes, and soaps. Allergy testing was positive to dust mites, all stinging insects except for honeybee, and dog dander. Her tryptase level was also elevated above normal (normal range, < 11 ng/mL). A timeline displaying the patient's major symptoms and path to diagnosis is shown in Figure 1.

BSC initiated in February 2018 included 2 different H1 antihistamines, montelukast (10 mg daily), cromolyn (200 mg 4 times daily), and epinephrine (0.3 mg as needed [PRN]). A hematologist specializing in myeloproliferative neoplasms was consulted in March 2018. Lab findings (test result; normal range) were notable for white blood cells (11.4 × 10^9^/L; 4.0–11.0 × 10^9^/L), hemoglobin (14.7 g/dL; 12.0–15.1 g/dL), platelet count (320 × 10^9^/L; 150–450 × 10^9^/L), basal tryptase level (14.1 ng/mL; < 11 ng/mL), alanine aminotransferase (62 U/L; 4–36 U/L), aspartate aminotransferase (51 U/L; 10–36 U/L), total protein (8.2 g/dL; 6.0–8.3 g/dL), lactate dehydrogenase (565 IU/L; 313–618 IU/L), and albumin (4.8 g/dL; 3.4–5.4 g/dL). She was negative for HIV, hepatitis C virus antibody, hepatitis B core virus antibody, and hepatitis B surface antigen. A BM biopsy showed an atypical MC proliferation and 60% cellularity with trilineage hematopoiesis. Flow cytometry identified an aberrant MC population that expressed CD2, CD25, and CD30 (Figure 1). An absence of dense MC aggregates was noted as not fulfilling the WHO diagnostic criteria for SM [14], despite morphologic and immunophenotypic features of the MCs being suggestive of the disease (Table 1) [14–16].

A *KIT* mutation was not detected by next-generation sequencing (NGS) mutation profiling. Karyotype was normal. The *FIP1L1-PDGFRA* fusion transcript was not detected. Missense mutation in exon 23 R882C with a variant allele frequency < 5% in *DNMT3A* was detected. Variants of uncertain origin in *RARA* exon 7 T275M and likely germline origin in *TET2* exon 3 P363L were noted. There was no T-cell receptor-γ gene rearrangement.

In April 2018, the patient developed randomly occurring urticaria outbreaks. Her omalizumab (300 mg) prescription for urticaria was switched to once every 2 weeks in December 2018, and ranitidine (150 mg twice daily [BID]) and albuterol PRN were added to her BSC. BM biopsy in January 2019, again, did not meet WHO criteria for SM (Table 1) [14–16]. No atypical MC population was noted by immunohistochemistry (tryptase, CD2, CD117). A minute population of CD2+ and CD25+ MCs (0.05% of all cases) was detected by flow cytometry.

Notably, she tested positive for the *KIT* D816V mutation by high-sensitivity allele-specific oligonucleotide polymerase chain reaction (PCR) (Figure 1), and her serum tryptase level was elevated (16 ng/mL) but remained below the 20 ng/mL threshold used as an SM minor diagnostic criterion. Prior to the availability of these results, she began imatinib (400 mg daily), which offered no significant benefit and was discontinued a month later. Prednisone (10 mg daily), diphenhydramine (25–50 mg PRN), and doxepin (100 mg daily) were added to her BSC regimen.

In March 2019, she reported epinephrine injection use roughly once every 2 to 3 weeks since her last visit, often requiring emergency care. She was unable to work or travel and had a greatly diminished quality of life due to the burden of her symptomatology and anaphylactic episodes that had been occurring for almost 2 years. Physical and emotional stress triggered some anaphylactic episodes. Cromolyn helped considerably with diarrhea and abdominal pain; diarrhea now occurred once every 48 h instead of once every few hours. Omalizumab improved flushing episodes. She reported an intermittent rash, but skin biopsies were not obtained. Two weeks later, she underwent esophagogastroduodenoscopy and colonoscopy; all biopsies were negative for MC involvement.

Cytoreductive therapy was considered clinically necessary and midostaurin, a multikinase inhibitor with KIT-inhibitory properties that is approved for advanced SM and known to show clinical benefits in this patient population, was available for off-label use [17]. The patient was started on midostaurin (100 mg BID) on March 20, 2019, and prednisone tapered off the following week. Midostaurin caused nausea that was countered with ondansetron (8 mg), which was switched to granisetron (1 mg BID) to avoid the side effect of headaches. She had an anaphylactic episode in April 2019 due to ingestion of strawberries. In May 2019, she reported feeling substantially better since starting midostaurin, and diarrhea was only occasional. Despite a few days of flushing, no additional episodes of anaphylaxis occurred. Throughout the next 2 months, she only reported 2 anaphylactic episodes. One episode was due to a Hymenoptera sting; she injected epinephrine thrice and went to the ED. The second began with throat discomfort due to perfume exposure; she injected epinephrine once and took antihistamines. She continued to get mild reactions to known triggers and was treated early to prevent worsening of the reaction. She had been off prednisone for 1 month.

Sporadic muscle cramps in her legs and arms and ∼3 weeks of increased flushing began in October 2019. She had not taken any medications specific to these symptoms but continued all BSC medications, as well as midostaurin and omalizumab. She also began venom therapy once weekly, but it did not improve symptomatology in any notable manner.

She had 3 anaphylactic episodes due to unknown triggers in October 2019 that were less severe than prior to midostaurin. An anaphylactic episode triggered by peppermint ingestion occurred in November 2019. Epinephrine was injected thrice, followed by convulsing. She was treated and discharged by a local ED the same day. Another anaphylactic episode occurred in December 2019 due to stress and perfume exposure. She became incoherent and unresponsive, but no epinephrine was used. She was evaluated at a local ED and treated conservatively with fluids and medications at the physician's discretion.

In April 2020, a pathologist confirmed ISM according to WHO 2016 criteria [14] from a BM biopsy (*KIT* D816V mutation-positive, CD2 expression by flow cytometry, and > 25% abnormal MCs; Table 1 [14–16] and Figure 1); B- and C-findings were not present. Due to persistent symptoms during the past several months indicating loss of response to midostaurin, including worsening anaphylaxis, subcutaneous interferon (45 mcg once weekly) was prescribed in June 2020 and titrated up to 180 mcg weekly by August 2020, while awaiting the availability of a clinical trial at our site. Interferon-alpha, in case series and case reports, has demonstrated activity in SM [17]. After a month of interferon overlap, midostaurin was discontinued. Anaphylactic episodes again improved with change in treatment. She continued doing well (fewer anaphylactic episodes, mild diarrhea) through September 2020, with the exception of 2 anaphylactic episodes requiring epinephrine. She also reported intermittent brain fog, and some right femur pain and bone pain in the fingers. Otherwise, there were no major complaints. Although oxycodone was reportedly helpful for bone pain, as of January 2021 she was still losing consciousness every day due to her ISM despite increased salt intake. Intravenous famotidine (80 mg BID) helped reduce loose stools.

She had another anaphylactic episode requiring epinephrine in April 2021 and was taken to the ED. She reported having had numerous similar experiences but frequently not going to the ED. She also reported rashes that typically resolved on their own; this had been previously attributed to petechiae, but her platelet count had been normal at this point. She also noted experiencing a fall with an anaphylactic episode. She reported epinephrine use in early July 2021 due to perfume exposure.

**Figure 2:**
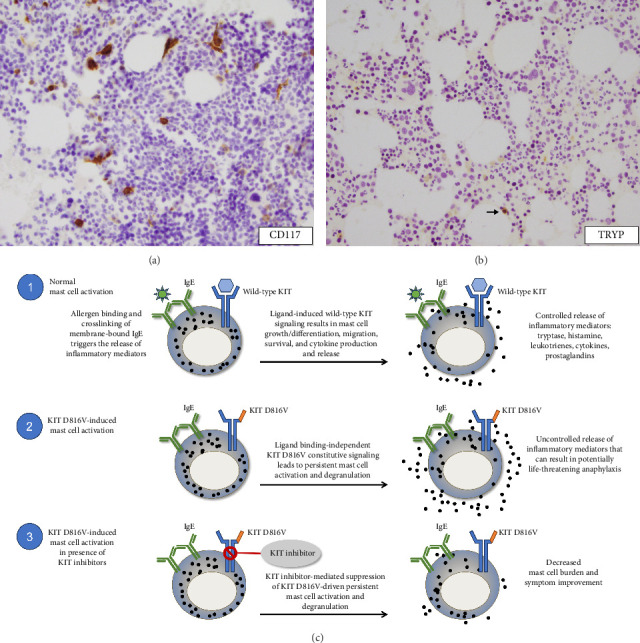
BM biopsies and role of KIT inhibition on mast cell activation. (a) BM biopsy at time of screening for clinical trial showing scattered interstitial mast cells that are predominantly spindle-shaped (CD117 immunohistochemical stain, 40×). (b) BM biopsy after 6 months of avapritinib treatment, which shows a rare interstitial mast cell (tryptase immunohistochemical stain, 40×) indicated by the arrow. (c) Schematic depicting mast cell activation in the presence and absence of *KIT* D816V mutation, and mechanism of action of KIT inhibitors. BM, bone marrow; CD, cluster of differentiation; IgE, immune-globulin E; TRYP, tryptase.

Avapritinib treatment (25 mg oral, daily) plus BSC was initiated in September 2021 as part of a clinical trial for ISM [18] (Figure 1). This potent and selective inhibitor of KIT D816V was not FDA approved at the time. BM biopsy, performed shortly prior to treatment initiation, was normocellular with trilineage hematopoiesis, and no dense MC aggregates were noted (Figure 2(a)). A mild increase of interstitial MCs was present (1% MC burden in BM biopsy) with 100% CD25-positive MCs and 90% spindle-shaped MCs (out of total MCs). Serum tryptase level was 12.1 ng/L, and *KIT* D816V was detected in the peripheral blood.

One anaphylactic episode triggered by perfume needed epinephrine in early October 2021. Through December 2021, the patient reported no major gastrointestinal symptoms or flushing, only mild anaphylactic episodes (one requiring epinephrine but no ED visit), and improved skin symptoms with better quality of life. There were no avapritinib-related side effects to this point, and omalizumab was discontinued.

In January 2022, she reported having an anaphylactic episode requiring 2 epinephrine injections, famotidine, and diphenhydramine, but no ED visit. She still experienced occasional, random episodes of postural orthostatic tachycardia syndrome. Otherwise, her symptoms had much improved relative to before treatment.

In February 2022, a BM biopsy showed no dense MC aggregates (Figure 2(b)). A mild increase in interstitial MCs (1% MC burden in BM biopsy) was noted with dim CD25-positivity and spindled shape in a subset of MCs (30% of total MCs). The BM continued to be normocellular with trilineage hematopoiesis. She reported doing well overall, although some skin lesions had begun to return and would worsen when showering. She continued to do well through April 2022, with no major changes. Her gastrointestinal and skin symptoms were diminished, and there was only one episode of anaphylaxis due to a known trigger.

In May 2022, she was bitten on the foot by an ant but did not develop any sustained local or systemic reactions. She did have a history of headaches and was diagnosed with idiopathic intracranial hypertension (opening pressure 40 mmHg). She started on acetazolamide 50 mg for 2 days, followed by 500 mg BID. The headaches soon became mild and intermittent. In August 2022, she had an anaphylactic episode requiring 2 epinephrine injections due to tomatoes. No episodes of anaphylaxis or epinephrine use were recorded since then, and through November 2022, she continued to do very well, with no anaphylactic episodes and only insignificant responses to triggers. Her cromolyn use has decreased, her diarrhea is infrequent and compared with February 2022, few skin lesions remain. Her acetazolamide dose was increased. Overall, she reported significantly improved quality of life and physical activity and is pursuing returning to work.

## 3. Discussion

The presented case highlights the diagnostic challenges and complexities in the management of patients with ISM. At the initial BM biopsy in 2018, our case presented features suggestive of SM, but no dense MC aggregates (major diagnostic criterion) were observed. She had also not yet exhibited cutaneous involvement, a ≥ 20 ng/mL serum tryptase level, or *KIT* mutation. Up to 40% of patients exhibiting recurrent, unexplained anaphylaxis without cutaneous symptoms have clonal MCs in BM without pathologic clusters [19], and approximately 30% of patients with SM have serum tryptase levels < 20 ng/mL [20]. Moreover, NGS lacks the sensitivity needed to detect *KIT*, leading to false-negative results when the MC burden in BM is low [13]. Hence, diagnostic delays are commonplace for patients with ISM and require a high index of suspicion from a physician familiar with mastocytosis as well as awareness of updated diagnostic criteria. Although the *KIT* D816V mutation was detected by allele-specific oligonucleotide PCR in the 2019 biopsy, our patient still only fulfilled 2 of the minor SM diagnostic criteria according to WHO 2016 guidelines (Table 1) [14–16]. The diagnosis of SM was made only in 2020 after 3 minor criteria were fulfilled.

Although < 5% of patients with ISM progress to advanced disease [13], they can experience life-threatening anaphylaxis and suffer from enduring physical and psychological symptoms that can heavily impact daily functioning and result in occupational loss and increased economic burden [21, 22]. The symptomatology and diminished quality of life of our patient were quite notable; especially considering both physical and emotional stress alone could trigger a severe anaphylactic episode. Compared with the general population, anaphylaxis occurs more frequently in adult SM, particularly ISM without skin involvement [9, 23, 24], and the most common trigger for severe anaphylaxis is Hymenoptera stings [24]. Our patient had to quit her job shortly after the initial Hymenoptera sting and even stopped leaving her home when possible due to her frequent, severe anaphylactic episodes typically triggered by specific foods, perfumes, and soaps.

Standard ISM management strategies include avoidance of triggers and symptomatic treatment with multiple therapies (over-the-counter and prescription). However, these types of BSC are largely ineffective, highlighting the need for the development of more effective, precision-based ISM-targeted therapies (Figure 2(c)). Our patient was diagnosed with *KIT* D816V mutation-positive ISM in 2020 based on WHO diagnostic criteria (Table 1) [14–16] while on treatment with midostaurin, a multikinase inhibitor effective against KIT D816V and approved for treating aggressive SM [25, 26]. Midostaurin was later discontinued due to loss of response, and about a year later, avapritinib treatment was initiated.

Avapritinib is a highly selective tyrosine kinase inhibitor for KIT D816V-mutant protein [18, 27] that has been found to significantly reduce symptoms and improve biomarkers of MC burden and quality of life in patients with ISM and advanced SM [18, 28]. Although individual results may vary, our patient experienced considerable symptomatic relief with no adverse drug reactions after ∼1 month of avapritinib treatment. Within 2 months of treatment, she was able to go outside again and play tennis, which she had been unable to do in a long time. Anaphylactic episodes (known and unknown triggers) became milder and infrequent, reducing the need for epinephrine and other supportive care drugs. After receiving avapritinib for more than a year, she was even able to go on a field trip with one of her children, which she was previously unable to do. She tolerated avapritinib well and remains on treatment.

## 4. Conclusion

The diagnosis and optimal management of this complex and rare disease require a multidisciplinary approach combined with effective personalized therapeutic strategies. Here we report a case of a patient who suffered repeated life-threatening anaphylactic episodes that greatly decreased her quality of life and that BSC measures failed to keep under control. A confirmed diagnosis of ISM led to the patient receiving what is currently an approved targeted treatment, with a favorable outcome. Novel precision therapies and refined diagnostic guidelines are critical to meet the high unmet needs of patients with ISM and improve their quality of life.

## Figures and Tables

**Table 1:** Diagnostic criteria for systemic mastocytosis [14–16].

	WHO 2016 [14]	WHO 2022 [15]	ICC 2022 [16]
Major criterion	Multifocal dense infiltrates of MCs (> 15 MCs in aggregates) in BM biopsies and/or sections of other extracutaneous organ(s)		Multifocal dense infiltrates of tryptase- and/or CD117-positive MCs (≥ 15 MCs in aggregates) detected in sections of BM and/or other extracutaneous organ(s)^a^

Minor criteria	*KIT*-activating point mutation(s) at codon 816 in the BM or another extracutaneous organ	Any *KIT* mutation with evidence of transforming behavior in the BM, blood, or another extracutaneous organ^a^	
	MCs in BM, blood, or another extracutaneous organ express CD2 and/or CD25	MCs in BM, blood, or another extracutaneous organ express CD2, CD25, and/or CD30	
	> 25% of all MCs are atypical type I or II cells on BM sections/smears or are spindle-shaped in MC infiltrates detected in sections of BM or other extracutaneous organs		
	Baseline serum tryptase > 20 ng/mL, unless there is an unrelated myeloid neoplasm, in which case this parameter is not valid^b^		

Diagnostic requirements	1 major + 1 minor criteria *OR* 3 minor criteria		1 major criterion *OR* 3 minor criteria

Abbreviations: BM, bone marrow; CD, cluster of differentiation; ICC, International Consensus Classification; MC, mast cell; WHO, World Health Organization.

^a^In the absence of a *KIT* mutation, the presence of tyrosine kinase gene fusions associated with myeloid/lymphoid neoplasm with eosinophilia and kinase gene fusion (M/LN-eo) must be excluded.

^b^If hereditary α-tryptasemia is also present, the tryptase level should be adjusted.

## Data Availability

The data that support the findings of the study are available from the corresponding author on request.
